# Paroxysmal Atrial Fibrillation in Females: Understanding gender diferences

**DOI:** 10.5935/abc.20180069

**Published:** 2018-05

**Authors:** Gabriel Odozynski, Alexander Romeno Janner Dal Forno, Andrei Lewandowski, Hélcio Garcia Nascimento, André d'Avila

**Affiliations:** 1 Universidade Federal de Santa Catarina - Florianópolis, SC - Brazil; 2 Serviço de Arritmia e Marcapasso - Hospital SOS Cardio - Florianópolis, SC - Brazil

**Keywords:** Arrhythmias,Cardiac, Atrial Fibrillation, Catheter Ablation, Cardiac Electrophysiology, Gender

## Abstract

**Background:**

The catheter ablation of atrial fibrillation (AF) is performed less frequently in women. In addition, there is divergent information in the literature regarding the effectiveness and safety for the ablative procedure to females.

**Objectives:**

The objective of this study was to compare the clinical characteristics and outcomes in men and women undergoing paroxysmal atrial fibrillation (PAF) ablation.

**Methods:**

Cohort study of patients undergoing first-ever PAF catheter ablation procedure refractory to antiarrhythmic drugs. The information was taken from patients’ records by means of a digital collection instrument and indexed to an online database (Syscardio®). Clinical characteristics and procedures were compared between each gender (M x F), adopting a level of statistical significance of 5%. The primary endpoint associated with efficacy was freedom from atrial arrhythmia over the follow-up time.

**Results:**

225 patients were included in the study, 64 (29%) women and 161 (71%) men. Women presented more symptoms due to AF according to the CCS-SAF score (1.8 ± 0.8M x 2.3 ± 0.8F p = 0.02) and higher CHADS2 score compared to men (0.9 ± 0.8M x 1.2 ± 1F). Post-ablation recurrence occurred in 20% of the patients, with no difference based on gender (21% M x 20% F p = 0.52). The rate of complications was less than 3% for both groups (p = 0.98).

**Conclusion:**

Women undergoing the first-ever PAF catheter ablation procedure present similar complication rate and clinical outcome compared to men. These findings suggest that the current underutilization of AF catheter ablation in women may represent a discrepancy in care.

## Introduction

Although age-adjusted prevalence of atrial fibrillation (AF) is relatively higher in men than in women, the absolute number of arrhythmia patients between genders is similar, with most cases occurring in patients between 65 and 85 years of age, a period in which, proportionately, more women are alive.^1^

Population studies show lower indication and execution rates of ablative treatment for AF in women with atrial fibrillation compared to men.^2^^-^^5^ However, it is not clear whether this represents a discrepancy in assistance or a real difference. Based on the assumption that higher rates of complications and recurrence occur in women compared to men, the underutilization of AF ablation in women in this case, could be understood as an appropriate difference and not a direct lack of assistance.

Previous studies evaluating differences between genders regarding safety and efficacy AF catheter ablation have conflicting results, and Brazilian literature on the topic is scarce.^6^^-^^13^ In this study, we evaluated clinical characteristics and outcomes of a current Brazilian women cohort undergoing ablation of AF per catheter compared to results obtained in men.

## Methods

### Study design and participants

Cohort study of patients undergoing the first catheter ablation procedure for paroxysmal atrial fibrillation (FAP) refractory to antiarrhythmic drugs with minimum follow-up time per patient of 12 months. The study was conducted between 2013 and 2015 in a single center. Information was collected from patients' records by means of a digital collect instrument and indexed to an online database (Syscardio®). Clinical characteristics and procedures were compared between genders (M x W). Primary endpoint associated with efficacy was atrial arrhythmia absence lasting > 30 seconds during the follow-up period after first and only ablation procedure.

### Procedures

All patients underwent circumferential isolation of pulmonary veins (PVs) through irrigated catheter ablation with a 3.5 mm tip without contact force measurement, using radiofrequency energy applications up to 35 Watts and 43ºC per 10-45 seconds and demonstration of entrance and exit electrical blockade of PVs in relation to left atrium at the insulation end. All procedures were performed under general anesthesia, orotracheal intubation and invasive blood pressure monitoring by radial or left femoral puncture by the anesthesiologist. Transseptal punctures were performed with the help of intracardiac Eco, which was maintained throughout the procedure. Applications to left atrium posterior wall were monitored by an oesophageal thermometer with multiple covered sensors (Circa) and stopped whenever there was a change in esophageal temperature above 38ºC. During all procedures, performed with an electro-anatomical mapping system based on thoracic impedance (EnSite Navx - Abbott), IV heparin bolus of 100mg / kg was performed followed by continuous infusion to maintain coagulation time activated between 350 and 450 sec.

After the procedure, patients remained on antiarrhythmic drugs (propafenone, sotalol or amiodarone depending on preference of attending physician) for 1 month and anticoagulant for a 3 months minimum period regardless CHA2DS2-VASc. It was done clinical follow-up 1, 3, 6 and 12 months after the procedure, performing ECG and at least two Holters throughout all the clinical follow-up. At the 10th week after ablation, patients were encouraged to perform continuous electrocardiographic monitoring (Holter) for 5 days. Any atrial arrhythmia greater than 30 seconds documented duration after 1 month of blanking period indicated arrhythmia recurrence.^14^ Symptoms severity before ablation and during eventual recurrences was characterized by the Canadian Cardiovascular Society Severity of Atrial Fibrillation score (CCS-SAF).^15^

### Statistical analysis

Clinical characteristics and procedures were compared between genders (M x W). Recurrence rates after a single procedure, as well as complications were also compared between groups. A convenience sample (non-probabilistic) was adopted during the study time, respecting the inclusion/exclusion criteria and follow-up time.

Continuous variables were described as mean and standard deviation and compared using unpaired Student's t-test (two-tailed), respecting the criteria of normality by the Shapiro-Wilk test. Categorical variables were described by absolute number and percentages in relation to total sample, and were compared using the c² test or Fisher's exact test. Level of statistical significance adopted was 5%. Kaplan-Meier curve was used to evidence recurrence rates on the follow-up time and the Log-Rank test to evaluate difference between groups (M x W). Statistical analysis was performed using IBM SPSS Statistics Editor software, version 22.0.

## Results

### Patients

225 patients undergoing AF ablation were included in the study: 161 (71%) men and 64 (29%) women. Regarding follow-up time, there was no difference between men and women. Table 1 summarizes clinical characteristics of men and women who underwent paroxysmal AF ablation during the study period. Regarding the mean age, women undergoing catheter ablation were older than men (57 ± 11 M x 62 ± 9 W p < 0.01) but there was no difference between groups in relation to body mass index (BMI) and left atrium anteroposterior diameter, although a smaller LV ejection fraction, possibly without clinical relevance, was observed among males (63 ± 10% M x 66 ± 6% W p < 0.05).

**Table 1:** Clinical characteristics of patients undergoing AF ablation, categorization by gender

Variables	Men (n = 161)	Women (n = 64)	p-value
Age (years)	57 ± 11	62 ± 9	0.001*
BMI	27 ± 3.7	27 ± 5	0.64
Ejection Fraction (%)	63 ± 10	66 ± 6	0.02*
LA Diameter (mm)	38 ± 5	38 ± 5	0.93
CHADS2	0.9 ± 0.8	1.2 ± 1	0.04*
CHF	12 (7%)	4 (6%)	0.73
SAH	85 (52%)	43 (67%)	0.06
Diabetes Mellitus	17 (10%)	11 (17%)	0.18
Coronary Artery Disease	25 (15%)	12 (19%)	0.44
Prior Stroke/TIA	6 (4% )	5 (8%)	0.06
CCS SAF score	1.8 ± 0.8	2.3 ± 0.8	0.02*
Statin Use	44 (27%)	26 (40%)	0.03
ACE/ARA-2 Inhib	66 (41%)	30 (46%)	0.25
Previous / current use of AA	134 (83%)	58 (90%)	0.21
Diagnostic time (months)	11 ± 12	14 ± 10	0.87
Follow-up time (months)	34 ± 17 (12 - 66)	33 ± 14 (13 - 64)	0.87

Values with ± indicate mean and standard deviation; CCS SAF: Canadian Cardiovascular Society Severity of Atrial Fibrillation scale; ACE: angiotensin converting enzyme; ARA-2: Angiotensin 2 receptor antagonist; Student t test and χ^2^ for independent samples.

* p-value indicates a statistically significant difference at the level of 5%.

There was also no difference between genders regarding comorbidities such as hypertension, diabetes mellitus, heart failure, coronary disease and previous history of stroke/TIA. However, women presented a higher CHADS2 score (0.9 ± 0.8 M x 1.2 ± 1 W, p = 0.04) and were more symptomatic than men according to the CCS-SAF score (1.8 ± 0.8 M x 2.3 ± 0.8 W p = 0.02). Between genders, there was no difference in the proportion of the use of ACE/ARA-2 inhibitors and antiarrhythmic drugs; however, women showed greater use of statins compared to men (27% M x 40% W p = 0.03 - Table 1).

### Efficacy and safety of procedures

Recurrence rates after single ablation procedure were similar between groups (21% M x 20% W p = 0.52). Table 2 summarizes procedures results as well as complications by gender. There were 3 inguinal pseudoaneurysms, 1 inguinal hematoma and 1 urethral trauma during bladder catheterization in men; among women, 1 inguinal hematoma and 1 retroperitoneal hematoma (5 (3%) M x 2 (3%) W p = 0.98) were observed. Despite prolonging hospitalization time, none of the complications required surgical intervention to be controlled. Throughout the study, atrium-esophageal fistulas, pericardial effusions, TIA/stroke after ablation or death were not reported.

**Table 2:** Results of the procedures: Efficacy and safety

Variables	Men (n = 161)	Women (n = 64)	p-value
Nº. of procedures	195	77	-
Complications *	5 (3%)	2 (3%)	0.98
Length of stay	2.5 ± 0.7 days	2.1 ± 0.8 days	0.76
Recurrence	34/161 (21%)	13/64 (20%)	0.89

Values with ± indicate mean and standard deviation;

* Men: 3 inguinal pseudo-aneurysms, 1 inguinal hematoma and 1 urethral trauma (bladder catheter). Women: 1 inguinal hematoma and 1 retroperitoneal hematoma; there were no deaths. Student's t-test and χ^2^. P-value indicates a statistically significant difference at the level of 5%

**Figure 1:**
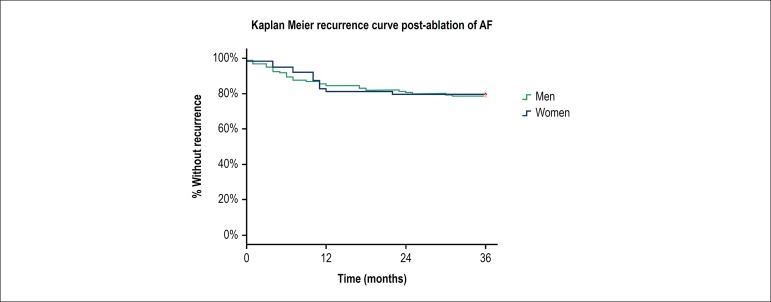
Kaplan Meier curves for clinical recurrence post ablation by catheter categorized by gender; Log Rank test for trend comparison between groups (MxW) p-value = 0.89

The Kaplan-Meier curve (Figure 1) shows, throughout the study, gender equity in relation to recurrence rates, which occurred more frequently in first 12 months of follow-up, regardless of patient's gender. There was no difference in patients hospitalization time (days) categorized by gender (2.5 ± 0.7 M x 2.1 ± 0.8 W p = 0.76).

## Discussion

Gender-specific differences may influence clinical and therapeutic behaviors in women with AF assistance. In a Canadian study, Singh et al.^16^ characterized safety and efficacy equivalence and homogeneity of ablative procedure between men and women with persistent AF (post-hoc MAGIC-AF Trial),^16^ guaranteeing its safety. In present study, in a current patients cohort with paroxysmal AF undergoing the first catheter ablation procedure, it is suggested that recurrence rates and complications are independent of patient's gender. These findings indicate that possible clinical considerations about safety and efficacy of ablative procedures in women with AF may be the main cause of ablation underutilization in female patients.

Gender-related differences in cardiac rhythm pharmacological control are well described in literature. Women are more symptomatic by the CCS-SAF score and report a lower improvement in quality of life when submitted to drug treatment, compared to men.^17^ In addition, female patients have a higher toxicity and intolerability rate to antiarrhythmic drugs than men, being more prone to *Torsade de Pointes* and need for pacemaker implants due to drug induced bradycardia.^17^^,^^18^ Therefore, catheter ablation can be considered as an early alternative for treatment of women with AF; it is a therapeutic method superior to drug therapy in maintenance of sinus rhythm^19^ with low complications rates in same proportion than men.

It is speculated that there are biological differences in the mechanism of AF between men and women, that, in theory, could justify different results when they undergo ablation, but such hypothesis seems unlikely. In previous studies, Walters et al.^20^ demonstrated left atrium and pulmonary veins electrophysiological characteristics similarity in men and women.^20^ Similarly; Pfannmuller et al.^21^ verified that there were no specific differences between genders due to atrial remodeling in AF through the expression of amyloid, collagen or bound junctions.^21^

In our study, the hypothesis that women in advanced age with AF present greater atrium electrical and structural remodeling and, consequently, worse post-ablation outcome, was not validated. The group of women was older than men and yet the time of diagnosis of arrhythmia is similar in both genders. In addition, left atrial diameter, a marker for post-ablation clinical recurrence, stroke, and death,^22^^,^^23^ was similar in both groups. The fact that same clinical outcomes were observed in the long term between the groups also suggests that, in our study, there were no significant biological differences between men and women undergoing AF ablation.^24^

### Limitations

In addition of being retrospective, the sample size may not have been sufficient to show differences between groups (M x W). The existence of selection bias in our cohort should also be considered, since only female candidates submitted to ablation procedure were included in the analysis. Finally, a detailed analysis was not performed in the evaluation of symptoms resulting from AF; instead, the CCS-SAF score was used comprehensively.

## Conclusion

In conclusion, in this population, women undergoing first AF catheter ablation procedure present clinical results regarding procedure safety and efficacy similar to men.
